# Comment on: “Investigation of intermediate CAG alleles of the HTT in the general population of Rio de Janeiro, Brazil, in comparison with a sample of Huntington disease‐affected families.”

**DOI:** 10.1002/mgg3.1243

**Published:** 2020-04-06

**Authors:** Gustavo L. Franklin, Alex T. Meira, Carlos H. F. Camargo, Hélio A. G. Teive

**Affiliations:** ^1^ Movement Disorders Unit Neurology Service Internal Medicine Department Federal University of Paraná Curitiba Paraná Brazil

We read with great interest the manuscript by Apolinário and colleagues (Apolinário, da Silva, Agostinho & Paiva, 2020) about the Investigation of intermediate CAG alleles of the HTT in the general population of Rio de Janeiro, and although we understand that was not the aim of the article, we would like to contribute with an interesting clinical finding, of an ongoing study of a southern Brazilian cohort, that may highlight the discussion about the importance of intermediate alleles in Huntington's disease (HD).

In a group of symptomatic patients with HD (OMIM: 143100) in a tertiary hospital in Brazil, two patients among a total of 41 patients evaluated were observed to present a classic HD phenotype. But interestingly, both of them had genetic CAG expansion at the intermediate alleles (IA) range—a mother and her son, with 29 and 34 CAG repetitions, respectively. Both patients had chorea, dystonia, and classical features of HD, indistinguishable from other patients in the classical CAG expansion.

In conformity with those findings, there is a growing scientific support of IA patients presenting a classical HD phenotype (Andrich et al., 2008; Cubo et al., 2016; Savitt & Jankovic, 2019; Squitieri & Jankovic, 2012). The probability of IA to manifest HD is of extremely relevance, not only for the pathogenesis comprehension of disease, but also for clinical and genetic counseling, since those individuals are often reassured as having no chance of developing HD (Squitieri & Jankovic, 2012).

Due to the expansion of CAG repeats greater than 26 CAG repeats, the HTT (4p16.3) gains an extra polyglutamine tail at the N‐terminal region, and once expanded HTT can be cleaved into fragments by proteases such as calpains and caspases. These protein fragments accumulate in specific regions as the medium spiny neurons inside nerve cells causing neuronal toxicity (Reilmann, Leavitt, & Ross, 2014).

The explanation of why some individuals with IA exhibit clinical symptoms while others do not is still controversial. A plausible justification is the presence of somatic mosaicism, and individuals with IA that manifest a HD phenotype, express longer CAG repeats in their medium‐striatal neurons than in other tissues in the same individual (Leija‐Salazar, Piette, & Proukakis, 2018). Mosaicism for CAG repeat length has been reported in CNS (Telenius et al., 1994). Therefore, age of onset and progression may depend also on other biological or environmental factors (Squitieri, Sabbadini, & Mandich, 2018; Wexler, Lorimer, & Porter, 2004), and there are studies of candidate gene modifiers that may influence age at onset and progression of the disease (Li, Friedman, & Li, 2007). Also, it may be the same reason of why not all subjects in the range of 35–39 repeats manifest symptoms. It has been estimated that patients in the RP range to have a 60% chance of being symptomatic at age of 65 years (Quarrell et al., 2007).

It is essential that the classification corresponds to the clinical reality, so that genetic counseling and, even more, medical care can be done correctly. Since not considering the actual range of CAG repetitions in which HD may manifest, we may deprive the patient of adequate follow‐up, and even making the treatment more distant, once new treatments are emerging.

It is likely that patients who express 27–35 CAG repeats have an even lower penetrance than those with 36–39, but the possibility of IA individuals producing a classic HD phenotype seems undeniable.

In this way, we may suggest that the IA range might belong to the same group of reduced penetrance range of HD, and we understand that a brand new genotypic and phenotypic classification of HD is imminent.

## CONFLICT OF INTEREST

The authors declare that they have no conflict of interest.

## AUTHOR CONTRIBUTIONS

GLF, ATM, and HAGT contributed to the initial development of the research, drafting, and review of the final manuscript. GLF takes responsibility for its overall content. All authors have read and approved the final manuscript.

## FUNDING INFORMATION

No funding to declare.

## ETHICS APPROVAL AND CONSENT TO PARTICIPATE

The study was approved at the ethics committee by number: CAAE 67,130,217.1.1001.0096. Written informed consent for participating in this study was obtained from all patients.

## Data Availability

All data generated or analyzed during this study are included in this published article.
